# The very low-frequency band of heart rate variability represents the slow recovery component after a mental stress task

**DOI:** 10.1371/journal.pone.0182611

**Published:** 2017-08-14

**Authors:** Harunobu Usui, Yusuke Nishida

**Affiliations:** 1 Department of Rehabilitation, AICHI Medical College, Kiyosu City, Aichi, Japan; 2 Department of Physical Therapy, International University in Health and Welfare, Narita City, Chiba, Japan; University of Rome Tor Vergata, ITALY

## Abstract

The very low-frequency (VLF) band of heart rate variability (HRV) has different characteristics compared with other HRV components. Here we investigated differences in HRV changes after a mental stress task. After the task, the high-frequency (HF) band and ratio of high- to low-frequency bands (LF/HF) immediately returned to baseline. We evaluated the characteristics of VLF band changes after a mental stress task. We hypothesized that the VLF band decreases during the Stroop color word task and there would be a delayed recovery for 2 h after the task (i.e., the VLF change would exhibit a “slow recovery”).

Nineteen healthy, young subjects were instructed to rest for 10 min, followed by a Stroop color word task for 20 min. After the task, the subjects were instructed to rest for 120 min. For all subjects, R-R interval data were collected; analysis was performed for VLF, HF, and LF/HF ratio. HRV during the rest time and each 15-min interval of the recovery time were compared. An analysis of the covariance was performed to adjust for the HF band and LF/HF ratio as confounding variables of the VLF component.

HF and VLF bands significantly decreased and the LF/HF ratio significantly increased during the task compared with those during rest time. During recovery, the VLF band was significantly decreased compared with the rest time. After the task, the HF band and LF/HF ratio immediately returned to baseline and were not significantly different from the resting values. After adjusting for HF and LF/HF ratio, the VLF band had significantly decreased compared with that during rest.

The VLF band is the “slow recovery” component and the HF band and LF/HF ratio are the “quick recovery” components of HRV. This VLF characteristic may clarify the unexplained association of the VLF band in cardiovascular disease prevention.

## Introduction

Heart rate variability (HRV) is used to measure autonomic activity. In 1996, a task force standardized the methods of HRV measurement [1]. One of the most commonly used methods for HRV analysis is power spectral density (PSD) analysis [2]. PSD analysis provides information on the frequency and amplitude of specific rhythms that exist in the HRV waveform [2]. The high-frequency (HF), low-frequency (LF), and very low-frequency (VLF) bands are extracted from the HRV signal, and the spectral power is calculated for each band [1]. The HRV analysis, particularly HF and LF/HF ratio, was widely used for analysis of autonomic nervous activity for cardiovascular diseases [3], stroke [4], and epilepsy [5,6]. The HF band indicates vagal activity; the LF/HF ratio indicates sympathetic activity [1,7]. The VLF band indicates sympatho-vagal balance and has unique characteristics. Studies have shown that the renin-angiotensin system (RAS) is related to the VLF band [7,8]. Furthermore, the VLF band has a stronger association with cardiovascular disease prognosis [9], metabolic syndromes [3], and all-cause mortality after traumatic brain injury than with the other HRV components [10]. In many studies, low VLF power has been associated with increased chronic inflammation [11–13], and the nocturnal VLF band may be a predictor of infection after acute stroke [14]. The VLF band is used as a predictor of prognosis [9,14–16]. Moreover, high VLF power is associated with a high exercise capacity [17]. However, the mechanisms of these VLF associations remain unclear.

Mental tasks have been used to investigate autonomic nervous system mechanisms [18,19]. Compared with resting conditions, HF and VLF powers decrease during the Stroop color word task [18], whereas the LF/HF ratio has been shown to increase [18]. After a mental stress task, the HF power and LF/HF ratio have been shown to immediately return to the original state [19]. These changes are referred to as “quick recovery.” However, the pattern of VLF change after a mental stress task is unknown. It has been reported that interleukin-6 (IL-6), which is a chronic inflammatory marker, increases after mental stress [20,21] and persists for more than 100 min [20,21]. Therefore, because the VLF band has been shown to be associated with IL-6 [13], VLF may also demonstrate a delayed recovery after mental stress.

We aimed to evaluate the characteristics of VLF band changes after mental stress. We hypothesized that the VLF band will decrease during the Stroop color word task, with a delay in recovery for 1–2 h after the task. We speculated that the VLF changes after a mental stress task would exhibit a “slow recovery.” These findings could help to clarify the mechanisms of the VLF component in the prevention of cardiovascular disease.

## Materials and methods

### Subjects

Overall, 19 healthy men participated in this study. The subject data are shown in Table 1. All subjects were in their 20s, with a mean age of 26.5 years, and were all volunteers. All of the subjects were nonsmokers and had no known cardiovascular diseases. The subjects were recruited from Omaezaki Municipal Hospital and Seirei Christopher University, Japan. All subjects provided written informed consents for participation in this study. Ethical approval was obtained from the Ethics Committee of Seirei Christopher University and Omaezaki Municipal Hospital.

**Table 1:** Data of analyzed subjects.

**Age (years)**	26.5 ± 4.2
**BMI (kg/m^2^)**	23.3 ± 2.6
**HR (beats per minute)**	70.9 ± 10.9
**lnHF**	5.5 ± 0.7
**lnLF/HF**	1.3 ± 0.5
**lnVLF**	7.9 ± 0.9

Values are expressed as mean ± standard deviation.

BMI = body mass index; HR = heart rate; ln = natural logarithm; HF = high-frequency component; LF = low-frequency component; VLF = very low-frequency component.

### Protocol

This study was performed at Omaezaki Municipal Hospital in the occupational therapy room, which was quiet and bright and had a constant temperature of 22–24°C. The subjects were seated in chairs during the protocol. The subjects were first instructed to rest for 10 min (i.e., REST time), followed by a Stroop color word task for 20 min (i.e., STROOP time). After the task, the subjects were instructed to rest for 120 min (i.e., RECOVERY time). For all subjects, R-R data were measured to assess HRV from rest to recovery. A heart rate monitor (RS800/Polar/Finland) was used for recording and processing of the R-R data. The Stroop color word task was used to increase psychological stress. In previous studies, the Stroop color word task has been shown to induce increased psychological stress, as indicated by increased cortisol and increased sympathetic activity [18,22–26]. In the Stroop color word task, the Japanese words for “red,” “green,” “blue,” or “yellow” were shown on a computer screen in a random order. The letters appeared in a color different from that spelled by the word. Subjects were asked to say the color of the letters as quickly and correctly as possible. The colored words were presented randomly every 0.75 s for 20 min.

### Heart rate variability

The heart rate monitor was placed on the xiphoid process. The RS800 heart rate monitor recorded the heart rate by detecting the R wave. The heart rate monitor automatically calculated the RR interval. The RR interval data were transferred to a personal computer for further analysis. The program automatically removed any abnormal RR signals from the analysis. MemCalc/Tarawa software was used to perform HRV PSD analysis. Spectral power was calculated within each frequency interval using the following parameters: VLF power = 0.003–0.04 Hz; LF power = 0.04–0.15 Hz; HF power = 0.15–0.40 Hz; and LF/HF ratio. Due to skewed distributions, VLF, HF, and LF/HF ratio were transformed by natural logarithms (ln). All HRV values are expressed as mean ± standard deviation. The mean REST and STROOP time HRV data were calculated during each time period, and the mean RECOVERY time HRV data were calculated every 15 min.

The HRV analysis is affected by noise. We inspected these noises and ectopic beats and edited out the HRV data as recommended by the task force [1]. In task force, a window length of more than 5 min for VLF identification is recommended [1]. The software MemCalc/Tarawa combined with the maximum entropy spectral analysis could analyze VLF in less than 5 min. Additionally, we used the data just central 5 min in each period of the study. Therefore, the window length of VLF is considered appropriate.

### Statistical analyses

A paired t-test was performed to compare REST and STROOP time HRV. Dunnett’s test was performed to compare REST HRV and HRV at each 15-min RECOVERY time interval. The relationship between the VLF component at REST and at each 15-min RECOVERY time interval was affected by the HF and LF/HF components. VLF showed slow recovery, whereas HF and LF/HF ratio showed quick recovery. Therefore, analysis of covariance (ANCOVA) was used to adjust for confounding variables, i.e., the HF and LF/HF components, at each time point. Within-time differences were tested using Bonferroni’s inequality after ANCOVA. In all analyses, two-tailed tests were performed, and a p-value of <0.05 was considered to be statistically significant. The analyses were performed using the SPSS software (version 19.0; SPSS, Inc, Chicago, IL).

## Results

All of the subject data were utilized. Table 2 shows HRV changes during the STROOP time compared with that during the REST time. Compared with the REST time, the STROOP time HF and VLF components of HRV were significantly decreased. The STROOP time LF/HF ratio was significantly increased compared with the REST time (p < 0.05).

**Table 2:** HRV changes during Stroop color word task.

HRV component	Rest	Stroop
lnHF	5.53 ± 0.7	5.18 ± 0.17*
lnLF/HF	1.32 ± 0.54	1.80 ± 0.53*
lnVLF	7.87 ± 0.94	7.13 ± 0.58*

Values are expressed as means ± standard deviation.

* p < 0.05 vs. rest (paired t-test).

ln = natural logarithm; HF = high-frequency component; LF = low-frequency component; VLF = very low-frequency component.

In contrast, comparison of the REST time HRV and the RECOVERY time HRV was different (Table 3). For each 15-min RECOVERY time interval, the VLF component was significantly decreased compared with the REST time VLF (p < 0.05).

**Table 3:** Change of HRV during recovery.

	Rest	Recovery (min)							
		15	30	45	60	75	90	105	120
lnHF	5.53 ± 0.69	5.44 ± 0.66	5.63 ± 0.75	5.57 ± 0.82	5.67 ± 0.74	5.62 ± 0.73	5.63 ± 0.71	5.74 ± 0.70	5.72 ± 0.69
lnLF/HF	1.32 ± 0.53	1.73 ± 0.44	1.64 ± 0.61	1.65 ± 0.63	1.67 ± 0.57	1.68 ± 0.63	1.72 ± 0.53	1.72 ± 0.50	1.63 ± 0.50
lnVLF	7.87 ± 0.91	6.75* ± 0.62	6.91* ± 0.45	6.82* ± 0.54	7.04* ± 0.70	7.24* ± 0.75	7.28* ± 0.72	7.39* ± 0.74	7.32* ± 0.59

Values are expressed as mean ± standard deviation.

* p < 0.05 vs. rest (Dunnett’s test).

ln = natural logarithm; HF = high-frequency component; LF = low-frequency component; VLF = very low-frequency component.

**Fig 1:**
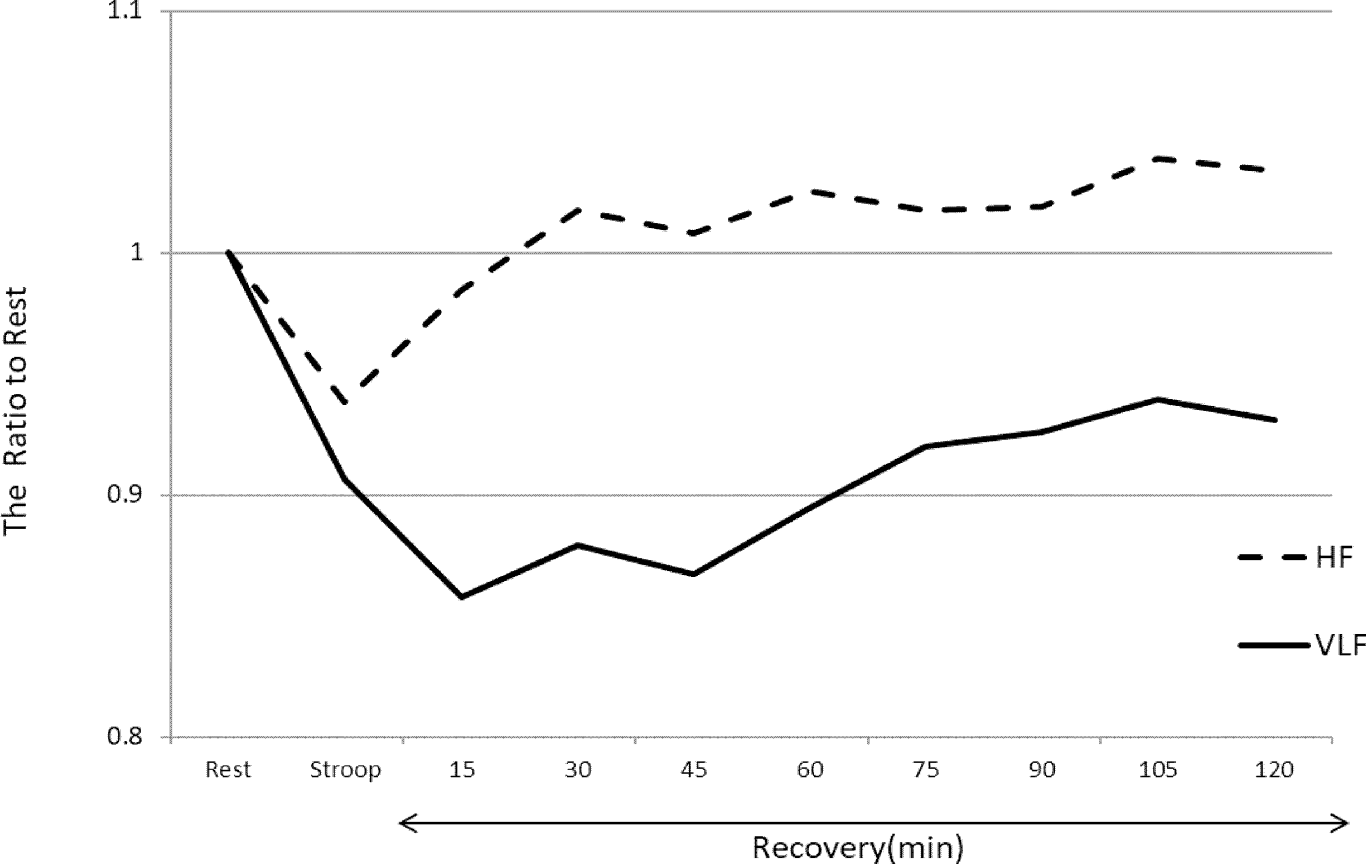
Ratio of Stroop and recovery to rest for HF and VLF components. HF = high frequency; VLF = very low frequency. This graph shows the HF and VLF component changes during the Stroop color word task and recovery after the task. The graph shows the ratio of Stroop and recovery to rest times. The solid line shows the VLF component of HRV, and the dashed line shows HF. During the Stroop color word task, both the HF and VLF components decreased from that during rest. In the recovery time, the HF component recovered to baseline a few minutes after the Stroop color word task; however, the VLF component remained decreased for 2 h after the task.

**Fig 2:**
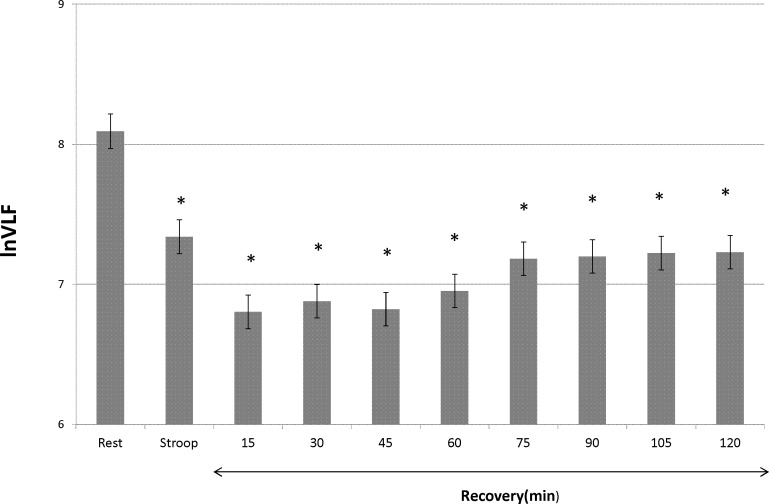
VLF changes adjusted for HF and LF/HF ratio. ln = natural logarithm; VLF = very low frequency; HF = high frequency; LF = low frequency. * p < 0.05 vs. rest (Bonferroni’s inequality). This graph shows the change of the VLF component during the Stroop color word task and for each 15-min recovery time interval to 120 min after the task. The VLF component was adjusted for HF and LF/HF ratio. The X-axis shows the VLF component after correcting by natural logarithm. The error bar shows the standard deviation. The asterisk indicates a significant difference from the rest value by Bonferroni’s inequality.

However, HF and LF/HF ratio did not significantly change. The differences in the slow and quick recovery components are described in Fig 1. Fig 1 shows the ratio of the STROOP and RECOVERY times to the REST time according to the HF and VLF components. The HF component immediately recovered to baseline after the STROOP time. In contrast, the VLF component did not recover in the 120 min after the STROOP time. After adjusting for HF and LF/HF ratio, each of the 15-min RECOVERY time periods of the VLF component was significantly decreased compared with the REST time (p < 0.05) (Fig 2).

## Discussion

The HF and VLF components of HRV decreased during STROOP time and the LF/HF ratio increased during STROOP time compared with that during the REST time (Table 2). These HRV changes observed in this study are similar to those observed in previous studies [18,19]. Accordingly, the Stroop color word task was sufficient as a mental task to activate the autonomic nervous system. Sympathetic activity decreases the VLF band, and vagal activity increases the VLF band [7,8]. Decrease of the VLF band during STROOP time suggests that sympathetic nervous activity was activated and vagal activity was inhibited by the mental task. During the RECOVERY time, only the VLF band continued to decrease for 120 min compared with the REST time. Conversely, the HF band and LF/HF ratio during the RECOVERY time were not significantly different from the REST time (Table 3). As shown in Fig 1, the VLF band took time to recover to baseline; however, the other HRV components recovered quickly. Therefore, the HF band and LF/HF ratio represented the “quick recovery” component. In contrast, the VLF band represented the “slow recovery” component.

The slow recovery of VLF resembles the change in chronic inflammation, which has been confirmed in a previous study [20,21]. In that study, inflammatory markers (e.g., IL-6) were elevated during and after the mental task and remained high for a few hours after the mental task [20,21]. Why did the VLF band and inflammatory markers exhibit delayed recovery? Any mental task activates the RAS [18]. The RAS responds to stress stimulation and increases inflammation to activate the sympathetic nerves [27]. Angiotensin II increases sympathetic discharge and inhibits vagal tone [28]. Furthermore, angiotensin II prolongs sympathetic nervous activity to inhibit noradrenalin reuptake by stimulating the sympathetic ganglia [28,29]. In a past study on HRV mechanisms, the VLF band was shown to be associated with the RAS [8]. The slow recovery of the VLF band after mental stress may indicate that sympathetic activity was prolonged by the RAS after the mental task.

Sympathetic nervous activity increases the LF/HF ratio and decreases the HF and VLF powers [7]. Vagal nervous activity increases the HF and VLF powers and decreases the LF/HF ratio [7]. We propose that the HF band and LF/HF ratio are the “quick recovery” components, and the VLF band is the “slow recovery” component. We postulate that the VLF band remained high for 2 h after the mental task because it exhibited slow recovery. However, quick recovery components could influence the VLF component during the RECOVERY time. To investigate this, we performed ANCOVA with the HF band and LF/HF ratio as potential confounders. After adjusting for HF and LF/HF ratio, the VLF component during the RECOVERY time was not influenced by the quick recovery components (Fig 2). These data showed that the VLF component is an independent “slow recovery” component.

This study has several limitations. First, we did not measure inflammation and RAS markers. Therefore, the mechanism of slow recovery is unclear. Hence, further study is needed to clarify the mechanisms in detail. Second, reactions to stress have individual differences, and some subjects may have had a lot of stress, whereas other subjects may not have had stress during the RECOVERY time. The quick recovery components, i.e., the HF band and LF/HF ratio, did not change during the RECOVERY time. This suggested that the subjects were not stressed, based on the quick recovery component changes during the RECOVERY time.

In past studies on stroke patients, the VLF band has been associated with physical activity, but the HF band and LF/HF ratio have not [4]. The VLF band has characteristics that are different from the other HRV components. In this study, we revealed new characteristics of the VLF band. We propose that the VLF band is the slow recovery component. The VLF band is related to the prognosis of cardiovascular diseases [9]; however, the mechanism remains unknown. The “slow recovery” may provide some evidence for future studies regarding the association of the VLF band and cardiovascular disease prognosis, which may prove useful in the prevention of cardiovascular diseases.

## Conclusion

HRV is a measure of autonomic nervous system activity. The HF band has been used to measure vagal activity, and the LF/HF ratio was used as a measure of sympathetic activity. The VLF band has several unique characteristics; however, its underlying mechanism of action remains unclear. In this study, we investigated the different HRV component changes following a mental task. Our results revealed that only the VLF band exhibited delayed recovery, which lasted for 2 h following the mental task. Therefore, we propose that the VLF band is the “slow recovery” component of HRV and that the HF band and LF/HF ratio are the “quick recovery” components of HRV.
